# Recommendations from the Council of Residency Directors (CORD) Social Media Committee on the Role of Social Media in Residency Education and Strategies on Implementation

**DOI:** 10.5811/westjem.2015.5.25478

**Published:** 2015-07-02

**Authors:** David Pearson, Robert Cooney, Michael C. Bond

**Affiliations:** * Carolinas Medical Center, Department of Emergency Medicine, Charlotte, North Carolina; † Conemaugh Memorial Medical Center, Department of Emergency Medicine, Johnstown, Pennsylvania; ‡ University of Maryland School of Medicine, Department of Emergency Medicine, Baltimore, Maryland

## Abstract

Social media (SM) is a form of electronic communication through which users create online communities and interactive platforms to exchange information, ideas, messages, podcasts, videos, and other user-generated content. Emergency medicine (EM) has embraced the healthcare applications of SM at a rapid pace and continues to explore the potential benefit for education. Free Open Access Meducation has emerged from the ever-expanding collection of SM interactions and now represents a virtual platform for sharing educational media. This guidance document constitutes an expert consensus opinion for best practices in the use of SM in EM residency education. The goals are the following: 1) Recommend adoption of SM as a valuable graduate medical education (GME) tool, 2) Provide advocacy and support for SM as a GME tool, and 3) Recommend best practices of educational deliverables using SM. These guidelines are intended for EM educators and residency programs for the development and use of a program-specific SM presence for residency education, taking into account appropriate SM stewardship that adheres to institution-specific guidelines, content management, Accreditation Council for GME milestone requirements, and integration of SM in EM residency curriculum to enhance the learner’s experience. Additionally, potential obstacles to the uptake of SM as an educational modality are discussed with proposed solutions.

## INTRODUCTION

Social media (SM) is a form of digital communication through which users create online communities using interactive platforms to exchange information, ideas, messages, podcasts, videos, and many other types of user-generated content. Emergency medicine (EM) has embraced the healthcare applications of SM at a rapid pace and continues to explore the potential benefit for knowledge translation, content management and education.^1^–^5^ The Free Open Access Meducation (FOAM) movement has emerged as a worldwide digital community of learning and practice, harnessing the ever-expanding collection of SM-based content and functioning as a virtual platform for the dissemination of knowledge and education.^6^

SM use by EM residencies continues to grow rapidly with a near ubiquity in its use.^7^–^8^ Opportunities now exist to enhance the current EM curriculum both with traditional learning sessions such as didactics or simulation, or with asynchronous learning.^9^ Learners are engaging with social-networking sites for educational purposes, and learner satisfaction is typically high with this modality.^10^–^12^ SM allows learners to interact and collaborate with content generators outside the confines of physical space and time. Learners report that SM provides the opportunity for peer collaboration, enhanced communication, and complementary learning.^13^–^17^ Users of SM for education report a strong desire to use it but also a strong desire to maintain privacy, specifically between their personal and professional lives. At present, there remains a chasm between educators and residents on how to most effectively integrate and implement SM in their EM curriculum.

SM is not an essential modality for residency education; however, it has distinctive advantages for augmentation of residency curricula. SM helps generate discussion across institutions, allows for rapid, near-real-time peer review and exchange of ideas, increases the audience of a lecture or teaching curriculum, and can provide detailed analytics of viewership including information on the viewers’ location and time spent on the activity. Further, SM has helped numerous individuals advance their academic careers with additional academic opportunities (e.g. lecture opportunities, research projects, publications). SM also aligns with adult learning models of providing educational resources when and where the student is ready to learn. Finally, SM can decrease the time to knowledge translation as articles are often debated and discussed even before they reach the official print journal. Potential disadvantages to implementation of SM is over-reliance by learners over conventional educational modalities (e.g. textbooks, manuscripts), technological barriers decreasing widespread use, potential inaccuracies of posted content, and potential legal or professional risks associated with posting private health information or inappropriate content. With SM in education ever expanding, continuing to assess these advantages and disadvantages plays an important role in effective use of SM in medical education.

According to the Accreditation Council for Graduate Medical Education (ACGME), “milestones are knowledge, skills, and other attributes for each of the ACGME competencies organized in a developmental framework from less to more advanced.”^18^–^19^ SM is another modality to achieve milestones-based competency, ranging from professionalism to medical knowledge and proper use of SM may be viewed as another “entrustable professional activity.”^20^

The goals of this guideline statement are to focus on SM as a tool for residency education. These guidelines are designed to provide support for EM residency programs in the development and use of program-specific SM presence for residency education. They are designed to complement and do not supersede any institutional guidelines or local, state or federal laws. The SM guidelines outlined in this paper constitute an expert consensus opinion, from a group of current program directors, educators and SM contributors, for best practices of the use of SM in EM residency education. The scope of this document is to function as a foundation document for further development of more detailed content and location-specific guidelines and best practices.

## METHODS

In 2013, a SM Committee that reports to the Council of Residency Directors (CORD) Board of Directors was formed. The committee met regularly to review available literature and discuss best practices of integrating SM into their educational curricula. At the CORD Academic Assembly in 2014, a pre-day session entitled “#dontgetleftbehind: FOAMed and SM for EM Educators” further explored the use of SM in education. Due to the considerable variation among institutions regarding the use of SM in residency education, the committee developed a GME-specific set of recommendations. These recommendations formed the preliminary work of this manuscript.

After the preliminary stage, members of the forum and other identified experts in SM who have been recognized in the field for their use of SM for education purposes used a modified Delphi technique, specifically, a discursive method based on an online discussion platform (Google Groups) curated and moderated by the chairperson of the committee. Four rounds of comments and revisions were obtained before the authors reconciled the recommendations and edited the final document.

These recommendations were approved by the CORD Board of Directors.

### Recommendations

CORD supports the use of SM as a valuable GME tool. Each residency program is encouraged to create a presence on SM and build SM content to enhance the sharing of knowledge. The CORD SM Committee developed the following recommendations for best practices in use of SM in residency education.

1. Institutional support: It is our recommendation that each institution allow and encourage residencies to use SM in their educational efforts. Institutional officials should be involved in the development of policies for its use, including allowing access to SM within the hospital network firewall. Specifically, access to medical education content on SM sites during clinical shifts can enhance clinical education as it facilitates real-time learning and feedback.^11^ SM can also help augment educational discussions by providing additional resources, and allow the viewpoints of those who are not physically present in the conference.
2. Residency leadership: Residency leadership should discuss the institutional SM policy and guidelines with residents, and ensure SM is used for educational purposes in an appropriate manner. The residency leadership or a designee should be responsible for monitoring the content generated by learners or other users of the designated sponsored platform.
3. Residency education: SM is a digital tool for enhancing EM education, and leadership should integrate it into the curriculum. SM modalities such as blogs, podcasts, vodcasts, Twitter, and Google Hangouts have been successfully implemented into curriculum.^8^,^14^ Program directors and residency leadership should explore the various aspects of their curriculum to determine the best fit for their residency. For examples, see Figure. Additional goals of integration are to identify and curate SM as a means for facilitating lifelong continuing education, practice-based learning, professionalism, responsible communication, and the giving of medical advice. CORD’s SM Committee is currently developing an educational series on the use of these various modalities.
4. Posting for purposes of education: Descriptions of specific patient encounters and clinical images should not be posted to personal or public SM sites unless intended for the purpose of education and all patient identifiers have been removed. Protected health information, including photographs, may not be placed on personal or public SM sites without specific informed written consent given by the patient for this specific purpose. For private, secure, or protected internal SM sites, local institutional guidelines should be used.
5. Affiliation with institution/program:
  - The name and logo of the program or institution should only be used on the designated sponsored platform, unless specific written consent is obtained from the program or institution. They should not be attached to personal accounts, home pages or identifiers on SM, unless approved by the program or institution. If permitted, institutional public relations/marketing guidelines must be followed and are expected to be consistent with the professional standards of conduct of these institutions.
  - When a connection to the program or institution is apparent, it should be clearly stated that the content posted is that of the individual and not an official opinion or endorsement of the institution. The medical information contained in the SM platforms must have a clear disclaimer that the information is general, may not be applicable for specific patients, is provided for didactic purposes only and does not establish a clinical or legal standard of care.
6. Content management: For residency-sponsored SM sites, the program should designate a non-resident content manager who is responsible for oversight and compliance. It is also essential to provide appropriate attribution and citation to content posted online. Despite the relative ease by which information can be obtained digitally, content generated by other individuals in SM should be appropriately cited and referenced to the original online postings.^21^
7. Professional SM engagement: Patient privacy also must be maintained. This includes complying with institutional policies regarding patient privacy, non-discrimination, and anti-harassment. Breaches of privacy, confidentiality, professionalism, or abuse of coworkers through disparaging comments are not acceptable in any circumstance. Potential conflicts of interest should be disclosed. The use of sound judgment and accuracy in communication is paramount. Inaccuracies should be promptly corrected. Whenever possible, a citation or reference with posted educational information is encouraged and promotes academic integrity.
8. ACGME milestone assessment: SM can be used as a tool to address several milestones. Specifically, EM milestones 15, 18, 19, 20, and 21 that address medical knowledge, technology, practice-based performance improvement, professional values, and accountability, respectively.

## CHALLENGES

Implementation of SM as an educational tool has some potential obstacles.^15^,^17^ Some of these obstacles are:

1. Hospital system/administrative barriers: Despite the growth of SM in medical education, some institutions still do not allow access to SM sites on their institutional networks or limit the participation of its employees on them. Reasons of limiting access include the possibility of decreased productivity, and the risk of exposing the institution if there are unprofessional posts. The hospital’s information technology (IT) teams should be involved early. Multiple departments within an institution (i.e.: IT, legal, public relations) will need to be involved for SM access approval via hospital computers, as well as to establish a residency SM presence. It is recommended to provide a proposal that outlines your purpose for education and plan for oversight and monitoring.
2. Technology barriers: Providing educational sessions to faculty and residents is strongly recommended to familiarize them with the technology. This can be provided in conjunction with intern orientation, faculty meetings, and educational conference.
3. Engagement and time constraints: The use of SM in a curriculum requires a committed faculty and dedicated educators to create the educational experience via SM to optimize the learner’s experience. Several recurring themes are evident that may create faculty reluctance to incorporating SM and FOAM.
  - Additional funding and resources required to integrate into curriculum: As SM integration can require extra work over existing educational modalities, educators can use pre-existing FOAM materials to supplement traditional approaches. Some institutions report that residents can help with early implementation to foster faculty engagement. Expenditures related to the use and creation of SM content can be thought of in terms of capital expenses and time expenses. Capital expenses can range from zero for free platforms (Facebook®, Twitter®) to several thousands dollars for independently hosted blog and podcast platforms. Time expenses are difficult to quantify. Faculty often create this content separate of their teaching responsibilities, however, utilizing these tools may also fit within protected time used to develop curricula and other learning resources.
  - Peer review on SM platforms: SM posts to the most popular SM education sites have post-publication peer-review with comments from a broad base of international medical practitioners. Some SM experts (www.aliem.com/pilot-aliem-journal/) are working to establish pre-publication peer-review processes on various SM platforms.^22^–^23^
  - Generational gap: FOAM continues to grow and many younger learners are using this to supplement their education. Integrating this into the curriculum is an effective means to ensure residents are receiving high-quality education and ensuring life-long learning strategies in the context of SM.
  - Information Overload: The explosive growth in the medical literature and number of available learning modalities has been present well before SM. FOAM allows for prioritization of topics of interest. However, it is important to be aware of the potential of overemphasis on certain “hot” topics.
4. Promotion and Tenure: Promotions and Tenure committees may currently not provide credit or incentives for educational content created on SM. However, SM has been used effectively to increase an individual’s national and international reputation, which can lead to a variety of academic opportunities (i.e. lecturing, manuscript authorship, committee participation).
5. Engaging learners: It is imperative to have diverse modalities to address adult learning. It is important to ensure SM enhances the broader learning modalities rather than be the sole tool. SM can augment the discussion of a journal article, blog post or educational podcast. Some residents are engaged in content curation on behalf of their program, which is another form of peer-to-peer learning.
6. Faculty endorsement of SM: In order to fully and effectively incorporate SM into a residency-wide curriculum one needs to have faculty and residency leadership that support SM as a legitimate form of education.
7. Barriers to privacy: The learner may have concerns over personal privacy. Strategies such as restricting viewership of personal posts can be used. Separating professional and private lives online can and should be done.
8. Quality assurance/Validation metrics: The quality assurance of blogs and tweets occurs with near-instantaneous post-publication review by other peer experts and are digitally responded to within minutes. Errors can be corrected and retracted in a short time period. Web-based technology can also provide high-quality validation metrics on the reach of SM. An author can obtain detailed metrics on viewership which includes location data (i.e.: country, state, city) and frequency of sharing.

## LIMITATIONS

Unfortunately, there are no scientific studies on the role of SM in residency education. Therefore, this manuscript is limited in that it represents the opinions and consensus of an expert panel and has all the typical limitations of expert panels.

## CONCLUSIONS

SM utilization in EM residency education continues to grow. Faculty and residents should receive education detailing appropriate SM stewardship that adheres to institution-specific guidelines. For residency program-sanctioned SM sites, the program should designate a content manager who is responsible for oversight and compliance. Institutions should endorse the use of SM as a part of medical education since access to high-quality educational materials can enhance the learner’s experience.

## DEFINITIONS

Program Leadership: Composed of the Program Director, Associate Program Director(s), Assistant Program Director(s) of an EM Residency Program, and the departmental Chair. Content Manager: Individual(s) in the organization entrusted with monitoring, contributing to, filtering, and otherwise guiding the SM presence of the program.

## Figures and Tables

**Figure:**
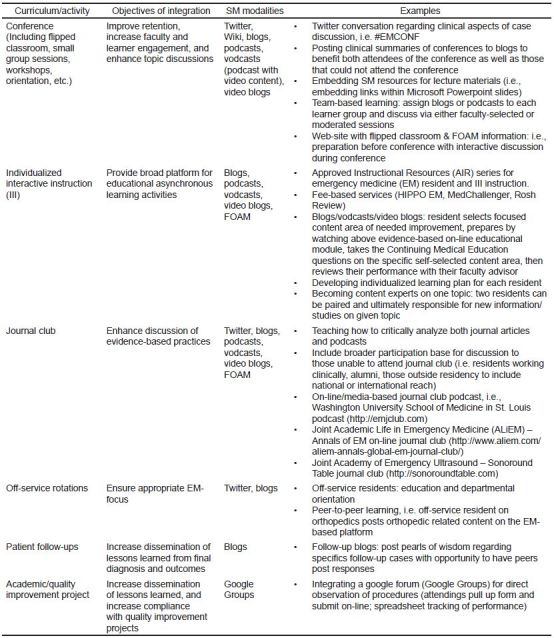

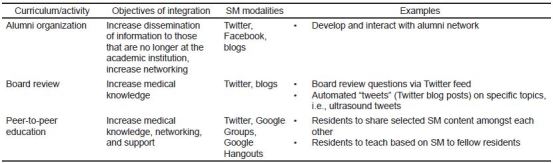
Examples of integration of social media into the emergency medicine curriculum. *SM*, social media; *EMCONF*, emergency medicine conferences; *FOAM*, free open access meducation; *HIPPO*, hippocrates
